# Mammography facilities serving vulnerable women have longer follow‐up times

**DOI:** 10.1111/1475-6773.13083

**Published:** 2018-11-05

**Authors:** Leah S. Karliner, Celia Kaplan, Jennifer Livaudais‐Toman, Karla Kerlikowske

**Affiliations:** ^1^ Department of Medicine Division of General Internal Medicine University of California San Francisco San Francisco California; ^2^ Multiethnic Health Equity Research Center University of California San Francisco San Francisco California; ^3^ General Internal Medicine Section San Francisco Veteran Affairs Medical Center San Francisco California; ^4^ Departments of Epidemiology and Biostatistics University of California San Francisco San Francisco California

**Keywords:** abnormal mammogram, breast cancer, delay, facility characteristics, vulnerable populations

## Abstract

**Objective:**

To investigate mammography facilities’ follow‐up times, population vulnerability, system‐based processes, and association with cancer stage at diagnosis.

**Data Sources:**

Prospectively collected from San Francisco Mammography Registry (SFMR) 2005‐2011, California Cancer Registry 2005‐2012, SFMR facility survey 2012.

**Study Design:**

We examined time to biopsy for 17 750 abnormal mammogram results (BI‐RADS 4/5), categorizing eight facilities as short or long follow‐up based on proportion of mammograms with biopsy at 30 days. We examined facility population vulnerability (race/ethnicity, language, education), and system processes. Among women with a cancer diagnosis, we modeled odds of advanced‐stage (≥IIb) cancer diagnosis by facility follow‐up group.

**Data Extraction Methods:**

Merged SFMR, Cancer Registry and facility survey data.

**Principal Findings:**

Facilities (N = 4) with short follow‐up completed biopsies by 30 days for 82% of mammograms compared with 62% for facilities with long follow‐up (N = 4) (*P* < 0.0001). All facilities serving high proportions of vulnerable women were long follow‐up facilities. The long follow‐up facilities had fewer radiologists, longer biopsy appointment wait times, and less communication directly with women. Having the index abnormal mammogram at a long follow‐up facility was associated with higher adjusted odds of advanced‐stage cancer (OR 1.45; 95% CI 1.10‐1.91).

**Conclusions:**

Providing mammography facilities serving vulnerable women with appropriate resources may decrease disparities in abnormal mammogram follow‐up and cancer diagnosis stage.

## 1 BACKGROUND

More than 200 000 U.S. women are diagnosed with, and approximately 40 000 die of, breast cancer annually.^1^ Disparities exist for ethnic minorities both for type of breast cancer diagnosed and across the care spectrum. This has been studied best and is most marked for African American women; however, particularly in the areas of disease stage, tumor characteristics, and processes of care, there are also considerable data to suggest differences for immigrant women and women living in poverty regardless of race‐ethnicity.^2^, ^3^, ^4^, ^5^, ^6^, ^7^, ^8^, ^9^, ^10^, ^11^, ^12^, ^13^, ^14^

Mammography can detect breast cancer that is not otherwise clinically detectable. For this reason, mammography has been broadly implemented as a means to achieve early detection and treatment of breast cancer.^15^ However, because mammography is not 100% specific and can only identify suspicious lesions, all abnormal mammogram results require either subsequent imaging or biopsy until the abnormality is defined as either cancer or benign.^16^ This additional evaluation must be done in a timely manner in order to avoid delays in cancer diagnosis and to have the potential to achieve the benefits of screening.^17^, ^18^, ^19^

While there is no definitive definition of the length of time between an abnormal mammogram and resolution that constitutes a delay, there is evidence that more than 3 months between presentation and treatment initiation can lead to more advanced cancer at diagnosis.^20^, ^21^, ^22^ Additionally, a recent simulation study reported that, with each additional 3‐month delay between an abnormal mammogram and subsequent diagnostic testing, the distribution of breast cancers shifted toward a higher stage.^19^ Delays in resolution of abnormal mammogram results can cause women psychological distress, including anxiety and depression.^23^, ^24^, ^25^ Multiple studies have found delays to be common, with estimates ranging from 20% to 40% of abnormal results having delayed follow‐up, and have delineated a disparity in which groups experience them: Delays are more prevalent for low socioeconomic status (SES) and minority women than for higher SES and White women.^3^, ^5^, ^26^, ^27^, ^28^, ^29^, ^30^, ^31^, ^32^, ^33^

However, examinations of the disparity in follow‐up care after an abnormal mammogram frequently emphasize the individual woman, her attitudes, intentions, and follow‐up behavior.^34^, ^35^, ^36^, ^37^ Two studies have observed that facilities serving vulnerable women have longer follow‐up times for abnormal mammogram results.^38^, ^39^ This demonstrates the need to assess processes of care at the facility level that may be contributing to timely or delayed follow‐up. While one study has demonstrated a relationship between communication processes and timeliness of follow‐up after an indeterminate mammogram result (Breast Imaging‐Reporting and Data System—BI‐RADS—0), no studies have investigated facility processes of care for more concerning BI‐RADS 4 or 5 results and follow‐up,^40^ or their relationship to disparities in follow‐up.

In this study, we examined whether facilities with longer follow‐up times for BI‐RADS 4 or 5 results serve a disproportionate number of vulnerable women compared with those with shorter follow‐up times and further examined facility characteristics associated with longer follow‐up times. Secondarily, we assessed whether stage of breast cancer diagnosis was associated with the facility's timeliness of follow‐up.

## 2 METHODS

### 2.1 Setting and data sources

**Figure 1:**
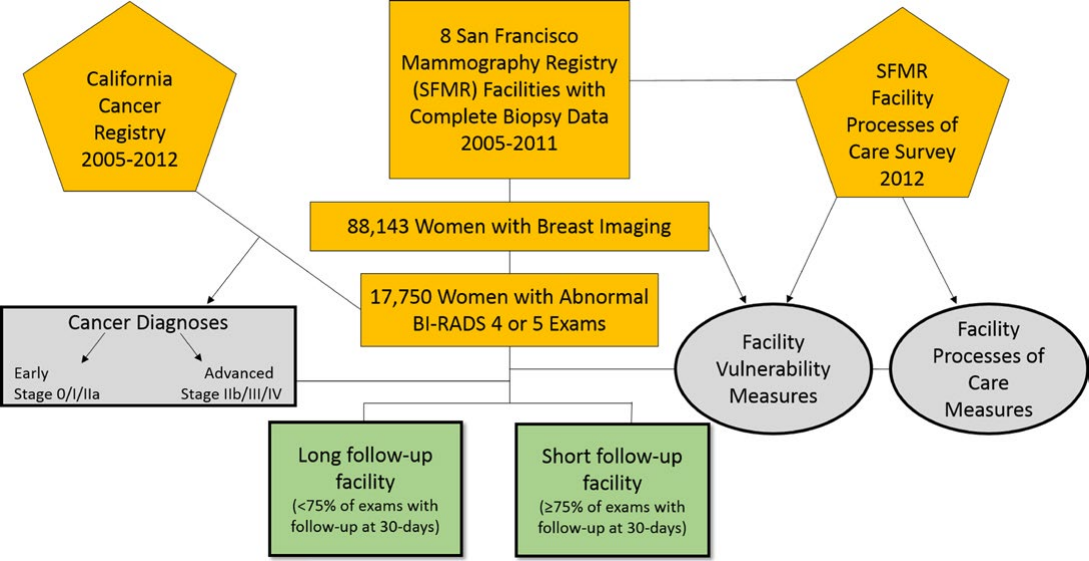
Sources of data and measure derivation from merged dataset [Color figure can be viewed at http://www.wileyonlinelibrary.com/]

This study is based on the San Francisco Mammography Registry (SFMR), a research registry of women having breast imaging at mammography facilities (“facilities”) in San Francisco and its surrounding counties (Figure 1).^41^ Facilities submit clinical data to the registry, including demographic information about women, mammography results, diagnostic examinations, and biopsy procedures for those women who give passive permission for their clinical data to be used for research. The SFMR annually links to the California Cancer Registry to collect information on cancer outcomes form the prior year.^42^

In 2012, we conducted a survey of the 13 facilities participating in the SFMR at that time to examine processes of care, including staffing, diagnostic appointment availability, tracking, communication practices with women and referring providers, and demographics of the population served not otherwise available in the SFMR (e.g., limited English proficiency [LEP]). We contacted the lead radiologist at each facility and asked them to indicate the best person—head technician or administrator—to participate in a survey about processes of care at that facility. Once identified, we contacted that individual by email and then set up a time in person or by telephone to administer the survey. For this analysis, we included data from the eight facilities with both complete facility and complete biopsy data in the 6 years prior to the facility survey (2005‐2011). These facilities were in San Francisco and Marin counties included an academic medical center, two public hospitals, and multiple community hospitals.

### 2.2 Measures

#### 2.2.1 Time to follow‐up

We defined time to follow‐up as the time between an index abnormal mammogram and biopsy. We included as index mammograms those done at one of the eight facilities between 1 January 2005 and 31 December 2011 with a BI‐RADS result of 4 (suspicious for malignancy) or 5 (highly suggestive of malignancy) and a recommendation for tissue biopsy.^16^ If a woman had multiple BI‐RADS 4 or 5 results, we included only the first one in the study period.

#### 2.2.2 Short vs long follow‐up facilities

We aggregated all of the examinations for each facility over the study period and plotted their mean time to follow‐up in days. Examining these plots, we found a clear separation in follow‐up at 30 days after the index mammogram, with half the facilities achieving biopsy follow‐up for at least 75% of abnormal BI‐RADS 4/5 mammograms at 30 days (range 75%‐95%) and the other half achieving follow‐up for less than 75% at 30 days (range 41%‐63%). Using 75% follow‐up at 30‐day cut‐off, we categorized each facility as having “short” (≥75%) or “long” (<75%) follow‐up.

#### 2.2.3 Population served by facility

Using the SFMR database for all women with any type of mammogram during the study period, we measured the distribution of race/ethnicity (African American, Asian, Latino, White) and educational attainment (<high school graduate, high school graduate/GED, some college, ≥college) for the population served by that facility. From the facility survey, we measured the proportion of the total population served with LEP. We then created three “vulnerable population” measures using a threshold of one standard deviation greater than the mean for all included facilities combined for that measure.^39^ The three vulnerable population measures were as follows: minority served (>59% African American, Asian, or Latina women), lower educational attainment served (>18% with <high school education), and LEP served (>36% with LEP). We then combined the three measures to create a vulnerable population served index (0 measures = none; 1‐2 measures = moderate; 3 measures = high).

#### 2.2.4 Facility characteristics and processes

Using the SFMR database for all diagnostic examinations regardless of result, we created a measure of examination volume (diagnostic examinations/week). From the SFMR facility survey, we measured staffing adequacy (number of full‐time‐equivalent or FTE radiologists reading per week), access (number of days to the next available biopsy appointment), tracking system (commercial integrated electronic vs homegrown spreadsheet or paper system), and communication of results (facility contacts provider directly beyond the report; facility contacts woman directly beyond the required letter; perception of who is primarily responsible for ensuring follow‐up: facility, referring provider, both).

#### 2.2.5 Cancer diagnosis

Using the merged SFMR‐California Cancer Registry data, we included any breast cancer diagnosis within 1 year of the index BI‐RADS 4/5 mammogram. Diagnoses were classified as early (0/I/IIa) or advanced stage (IIb/III/IV) according to summary stage available in the cancer registry.^43^

### 2.3 Analysis

We examined time to follow‐up for short and long follow‐up groups using a plot of cumulative percent and compared follow‐up rates at 30, 90, and 120 days using chi‐square statistics. We then compared facility characteristics, processes, and vulnerable population measures by short vs long follow‐up group using descriptive statistics.

Among women with a cancer diagnosis, we modeled the odds of being diagnosed with an advanced‐stage cancer according to having the index mammogram at a facility with short‐ vs long‐facility follow‐up. We further modeled those odds adjusting for individual characteristics known to be associated with cancer stage at diagnosis, including the woman's age, having a first‐degree relative with breast cancer, race/ethnicity, and the number of months from a prior mammogram to the index BI‐RADS 4/5 mammogram. In building our multivariate model, we accounted for clustering of patients within facilities, and we assessed the relative fit of model variations by comparing Akaike's information criterion values between models. We used a mixed effects model specifying facility as the random effect parameter and all other covariates as fixed effects.

## 3 RESULTS

### 3.1 Short and long follow‐up facilities

**Figure 2:**
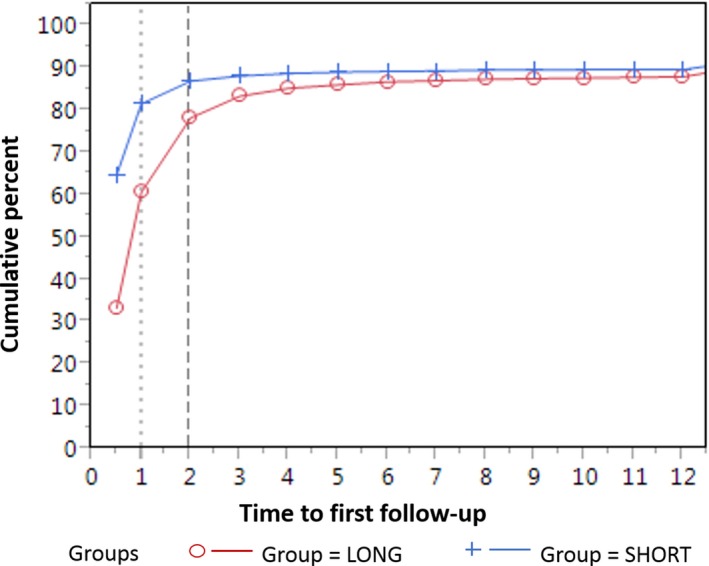
Time to biopsy after a BI‐RADS 4 or 5 mammogram result by short and long follow‐up facility group [Color figure can be viewed at http://www.wileyonlinelibrary.com/]

Our study sample included 17 750 index mammograms with BI‐RADS 4/5 assessment and recommendation for tissue biopsy follow‐up. The short follow‐up facilities achieved follow‐up at 30 days for a significantly higher proportion of mammograms compared to the long follow‐up facilities (82% vs 62.0%; *P* < 0.0001). The difference between the short and long follow‐up facilities was smaller but remained significant at 60 days (87.1% vs 78.5%; *P* < 0.001) and at 90 days (88.2% vs 83.7%; *P* < 0.001). The gap between the two groups was minimal by 120 days, or 4 months after the index mammogram, although the difference remained statistically significant (88.8% vs 85.4%; *P* < 0.001) (Figure 2). At 12 months, 10% of mammograms from short follow‐up facilities and 12% of mammograms from long follow‐up facilities did not have a documented biopsy in the SFMR.

### 3.2 Population served by follow‐up group

None of the short follow‐up facilities met criteria for any of the vulnerable population measures, whereas three of the four long follow‐up facilities met criteria for at least one of the measures, with two of those meeting criteria for all three measures indicating that they served a high proportion of vulnerable women (Table 1).

**Table 1:** Vulnerable population served and facility characteristics for San Francisco mammography registry facilities 2005‐2012 by facility short and long follow‐up group (N = 8 facilities)

	Short follow‐up facility group (four facilities; 15 203 mammograms)	Long follow‐up facility group (four facilities; 2547 mammograms)
Vulnerable population served (N, %)		
Minority served: >59% minority patients	0	3 (75.0)
Lower education attainment served: >18% patients with less than a high school education	0	2 (50.0)
Limited English proficient served: >36% patients with LEP	0	2 (50.0)
Vulnerable population served index		
None (0)	4 (100)	1 (25.0)
Moderate (1‐2)	0	1 (25.0)
High (3)	0	2 (50.0)
Volume, staffing adequacy, and access (mean, SD)		
Average diagnostic volume/week	87 (77)	12 (15)
FTE radiologists reading diagnostic examinations/week	5.5 (3.3)	3.3 (1.6)
Diagnostic volume/FTE ratio	250 (173)	379 (209)
Days to next available biopsy appointment	1.8 (1.3)	7.5 (6.8)
Tracking and communication (N, %)		
Tracking system		
Commercial	4 (100)	3 (75.0)
Homegrown	0	1 (25.0)
Facility contacts patient directly	4 (100)	2 (50.0)
Facility contacts physician directly	2 (50.0)	3 (75.0)
Responsibility for ensuring follow‐up		
Facility	3 (75.0)	0
Referring MD	0	1 (25.0)
Both	1 (25.0)	3 (75.0)

FTE, full‐time‐equivalent; LEP, limited English proficiency.

Within short follow‐up facilities, there was no difference in percent with follow‐up at 30 days by race/ethnicity (82% overall). However, within long follow‐up facilities, Black/African American women had the lowest percent with follow‐up at 30 days, followed by Chinese women (53% Black, 60% Chinese, 65% other Asians, 64% Latinas, 64% White; *P* = 0.03). Those with the lowest educational attainment (<high school) had a similar rate of follow‐up to the overall group in the short follow‐up group (81.8 vs 81.3; *P* = 0.76), but a slightly lower rate of follow‐up in the long follow‐up group (61.4 vs 63.2; *P* = 0.26).

### 3.3 Facility characteristics and processes

The long follow‐up facilities, on average, had lower volume of diagnostic examinations as well as fewer radiologists available to read those examinations, and a higher volume/FTE ratio than the short follow‐up facilities (Table 1). The long follow‐up facilities also reported longer waits for a biopsy appointment. While all but one of the eight facilities used a commercial tracking system, the two follow‐up groups differed in their approach to communication. All short follow‐up facilities, but only two of the long follow‐up facilities, reported that in addition to the legally mandated result letter, they contacted women directly to inform them of a BI‐RADS 4 or 5 result. While long follow‐up facilities reached out to the referring provider after a woman's BI‐RADS 4 or 5 result, they also perceived that the referring provider had equal or greater responsibility than the mammography facility for ensuring follow‐up was completed. By contrast, three of the short follow‐up facilities perceived that the responsibility lay primarily with the mammography facility and not with the referring provider.

### 3.4 Cancer diagnosis

There were 3099 cancer diagnoses in the 12 months following an index mammogram during the study period (Table 2). Of these, 480 were advanced stage (IIb or higher). A higher proportion of women with an advanced‐stage cancer (vs earlier stage) had no record in the dataset of a mammogram prior to the index mammogram (8.1% vs 3.5%; *P* = 0.000). Among women with a prior mammogram in the dataset, women with an advanced‐stage cancer had a longer time interval between the previous mammogram and the index mammogram (736 vs 652 days; *P* = 0.03).

**Table 2:** Stage of cancer diagnosis for cancers diagnosed after an abnormal mammogram at San Francisco mammography facilities 2005‐2012 by facility short and long follow‐up group (N = 3099 Women)

	Short follow‐up facility group (four facilities/2495 patients) N (%)	Long follow‐up facility group (four facilities/604 patients) N (%)
Cancer stage		
0	705 (28.3)	146 (24.2)
I	974 (39.0)	204 (33.8)
IIa	421 (16.9)	116 (19.2)
IIb	175 (7.0)	55 (9.1)
III(a,b,c)	150 (6.0)	50 (8.3)
IV	34 (1.4)	16 (2.6)
Missing	36 (1.4)	17 (2.8)

Women with an index mammogram at a long (vs short) follow‐up facility had 1.5 odds of being diagnosed at a higher breast cancer stage (OR 1.53; 95% CI 1.14‐2.05). This result was robust to adjusting for the woman's age, family history of breast cancer, race/ethnicity, and time between the previous mammogram and the index mammogram (OR 1.45; 95% CI 1.10‐1.91). Time between previous and index mammogram was marginally significant in the adjusted model (OR 1.01; 95% CI 1.00‐1.01).

## 4 DISCUSSION

We leveraged a unique dataset combining clinical data on thousands of women with abnormal mammogram results with information on the facilities serving those women to examine the relationship among follow‐up time, processes of care, and the vulnerability of the populations served by the facilities. We found that facilities serving a high proportion of vulnerable women—minorities, and those with low educational attainment or LEP—have long follow‐up time to biopsy for abnormal mammogram results suspicious for or highly suggestive of cancer. These facilities report processes of care that demonstrate fewer resources than their short follow‐up counterparts. They have less FTE radiologists reading diagnostic examinations, longer wait times for biopsy appointments, and less direct communication with women, with the expectation that the responsibility for this communication lies primarily with the referring provider. These findings point to the system processes that will need to be improved to address disparities in follow‐up for vulnerable women after an abnormal mammogram.

In comparison, within short follow‐up facilities, minority women and those with low educational attainment have rates of 30‐day follow‐up equal to White women and educated women. However, within long follow‐up facilities, Black and Chinese women, and women with less than a high school education have lower rates of 30‐day follow‐up than other groups. This indicates that not only are vulnerable women more likely to be served by lower resourced facilities, many of them fare worse than their White and more educated counterparts at those facilities. It also suggests that more resourced facilities are able to deliver equal care across groups regardless of race‐ethnicity or education.

For women diagnosed with cancer, getting initial care at one of these less resourced mammography facilities with longer follow‐up times after an abnormal mammogram is associated with a higher likelihood of being diagnosed with an advanced‐stage cancer. This is true regardless of race/ethnicity. In other words, Asian, Black, and Latina women diagnosed with breast cancer who received care at mammography facilities with short follow‐up times were less likely than their counterparts who received care at facilities with long follow‐up times to be diagnosed with an advanced‐stage breast cancer. This suggests that at least some of the disparity in cancer stage at diagnosis for minority women is related to the systems in which those women receive their care. This connection between disparities in place of care and an advanced stage of breast cancer at diagnosis drives home the imperative for addressing follow‐up procedures at the facility and health system level.

However, it may be that there are also individual factors contributing to this advanced‐stage diagnosis, some of which are unrelated and others related to follow‐up times. For example, unrelated to follow‐up, women with dense breasts and obese women are more likely to be diagnosed with advanced disease, and there are population differences for these factors.^44^, ^45^ Other individual and social factors likely play a role in follow‐up. In our sample, the advanced cancer group had a longer mean interval between a previous mammogram and the index abnormal mammogram, suggesting that the delay in follow‐up of the abnormal mammogram may not be the only delay contributing to an advanced cancer diagnosis. This prolonged interval itself may have multiple causes, including both individual and systems‐based reasons. Most previous examinations of the disparity in follow‐up care after an abnormal mammogram have emphasized the individual woman, her attitudes, intentions, and behavior.^34^, ^35^, ^36^, ^37^ However, the association we have found that vulnerable women are more likely to receive care from mammography facilities with long follow‐up belies the belief that the burden for timely follow‐up lies with the woman alone. Our findings highlight the need to improve care delivery and communication at the level of the mammography facility.

While there have been some successful interventions to decrease delays, these have largely focused on patient navigation in an attempt to address individual barriers to follow‐up.^46^, ^47^ While navigation programs have been successful, this personalized intervention guiding individual women through the complexities of breast cancer diagnosis does not seem to have its impact for at least 3 months after the abnormal mammogram.^48^, ^49^ Navigation's focus on the individual appears to be most successful for the patients at highest risk of nonfollow‐up, yet those patients are also the most challenging to achieve follow‐up for even in the context of a navigation program.^50^, ^51^ Thus, the focus of resource‐intensive navigation programs addressing individual barriers to follow‐up may need to be on the most at‐risk patients. However, there remains a need for system‐level solutions that can reach more women. Our findings suggest that there may be additional system‐based strategies specifically related to resources affecting staffing, access, communication, and coordination of care that facilities could employ to reduce disparities in follow‐up time and potentially cancer stage at diagnosis.

Our study has several limitations. Our data encompassed two counties in a single region, which may not be representative of other regions nationally. It is unclear exactly what is driving the variation in populations served by short and long follow‐up mammography facilities—preference, referral patterns, insurance coverage, neighborhood proximity, or other factors. We were not able to examine these factors in our dataset. Additionally, it is possible that a woman who had an index mammogram at one facility subsequently had her biopsy at another. While we were not able to capture biopsies performed outside of our eight facilities, we were able to capture biopsies done at any of the eight facilities included in the study regardless of the facility in which the index mammogram was performed. Finally, the time frame of our study only included the first years of the Affordable Care Act (ACA). Under the ACA, more traditionally underserved women have established primary care and now receive preventive services such as mammograms.^52^ This influx of newly insured women may have directed low SES women to both short and long follow‐up facilities; however, if the long follow‐up facilities do not have the resources to enhance their staffing, access, and communication capabilities, this could potentially worsen rather than lessen disparities.

In conclusion, we found that in our sample of mammography facilities, those serving high proportions of vulnerable women have long follow‐up times to biopsy, and their processes of care including adequacy of staffing, access, and communication practices differ from facilities with short follow‐up times. This places minority and lower SES women with breast cancer at higher likelihood of being diagnosed with advanced‐stage disease. Providing mammography facilities serving vulnerable women with appropriate resources may decrease disparities in abnormal mammogram follow‐up and cancer diagnosis stage.

## CONFLICTS OF INTEREST

The authors have no conflicts of interest to report.
